# Why should Medical Societies increasingly attend to their Specialist Title exams and why should medical professionals obtain it?

**DOI:** 10.1590/0100-6991e-20243750EDIT01-en

**Published:** 2024-04-22

**Authors:** GERSON ALVES PEREIRA, RAMIRO COLLEONI, LEONARDO EMILIO SILVA, LUIZ CARLOS VON BAHTEN, CÉSAR EDUARDO FERNANDES, PEDRO EDER PORTARI-FILHO

**Affiliations:** 1 - Universidade de São Paulo (USP), Curso de Medicina - Bauru - SP - Brasil; 2 - Universidade Federal de São Paulo (UNIFESP), Departamento de Cirurgia - São Paulo - SP - Brasil; 3 - Universidade Federal de Goiás (UFG), Departamento de Cirurgia - Goiânia - GO - Brasil; 4 - Universidade Federal do Paraná (UFPR), Departamento de Cirurgia - Curitiba - PR - Brasil; 5 - Associação Médica Brasileira (AMB), Presidente da AMB - São Paulo - SP - Brasil; 6 - Universidade Federal do Estado do Rio de Janeiro (UNIRIO) Escola de Medicina e Cirurgia - Rio de Janeiro - RJ - Brasil; 7 - Colégio Brasileiro de Cirugiões (CBC), Presidente do CBC - Rio de Janeiro - RJ - Brasil

**Keywords:** Societies, Medical, Professional Training, Quality Assurance, Health Care, Academic Performance, Sociedades Médicas, Capacitação Profissional, Garantia da Qualidade dos Cuidados de Saúde, Desempenho Acadêmico

## Abstract

Medical societies must maintain high standards of competence and quality when awarding specialist titles, defining the certification criteria, taking into account the needs and realities of the health system and medical practice.

## RESUMO

As Sociedades Médicas devem manter padrões elevados de competência e qualidade na concessão dos Títulos de Especialista, com definição dos critérios de certificação, considerando as necessidades e realidades do sistema de saúde e da prática médica.

## EDITORIAL

Competence and professionalism are nouns present in a doctor’s career with varied meanings and often used as opposites. Competence refers to knowing how to do things with discernment. Concurrently, professionalism combines technical competence with a continuous reflection of know-how, imposing an ethical sense on the physician’s actions.

Every competence happens in a certain context. Just as there is no authority for every field, it is also not conceivable to have a competence for every conceivable context. Thus, a physician recognized as competent in a technical understanding must adhere to the principles of bioethics, especially the principles of beneficence and non-maleficence.

However, the lack of technical competence preexists the lack of professionalism, but the existence of competence is not enough to denote technical competence.

In this scenario of ambiguity is the medical specialist who, in theory, means to society a doctor with professionalism preceded by a recognized technical competence relevant to his specialty.

The Specialist Title exam plays a vital role in promoting excellence and protecting patient health while contributing to the continuous advancement of the medical specialty. 

These examinations should be designed to ensure high standards of competence and professionalism among physicians seeking to become specialists. 

Societies must ensure the quality of the specialist title exam so that they can guarantee to the population that, by obtaining the specialist title, the physician has been entitled to the recognition of his technical competence, which is one of the highest and most important levels of medical training.

Article 17 of Law No. 3,268/57, which regulates the Councils of Medicine and gives other provisions, states that “The legally registered physician may practice his profession in any of its branches or specialties, assuming, of course, responsibility for his acts.”. However, Article 11 of the CFM Resolution prescribes that “It is forbidden for physicians and, where applicable, legal entities, trade unions, and medical associations to disclose, when not specialists, that they deal with organic systems, organs or specific diseases, by inducing confusion with alleged specialties”. 

In this sense, a specialist title from a medical society offers a variety of benefits, ranging from legal and ethical training, through professional recognition to continuous development, and participation in professional communities. 

These advantages can have a positive impact on both the surgeon’s career and satisfaction in his/her professional practice (Table 2). Obtaining a specialist title from a surgical society offers several advantages for a surgeon, both in terms of professional development and recognition in the medical field.

[Table t1]

**Table 1 t1:** Advantages of the Specialist Title for Medical Societies and the population.

Vantagens do Título de Especialista para as Sociedades Médicas e população	
Professional Competency Guarantee	It is one of the ways to ensure that surgeons who obtain certification possess the knowledge, skills, and competencies necessary to practice the specialty effectively and safely.
Patient Health Protection	By setting rigorous standards for certification, the surgical specialty society contributes to the safety and quality of patient care. Board-certified surgeons are recognized as professionals capable of delivering high-quality care.
Encourage the Ethical Practice of the Medical Act	By establishing the difference between moral conduct (right and wrong) and the reflection of one’s actions, which is ethics (good and evil). All of this refers the surgeon to the fundamental principles of our Code of Medical Ethics, especially to principle II: "The target of all the physician's attention is the health of the human being, for the benefit of which he/she must act with the utmost zeal and the best of his professional capacity."
Constant Update	The exam frequently covers recent advances in the surgical field, encouraging practitioners to stay up to date with the latest practices, technologies, and scientific evidence.
Specialty Standardization	Certification through the specialist title exam carried out by peers of the same specialty contributes to the standardization of practice in the specialty. This creates a common set of standards that benefits both practitioners and patients.
Recognition of the Specialization	It confers a formal recognition of the surgeon's ability in a specific area. This is valuable for the professional in terms of credibility and for society in ensuring that experts are properly recognized.
Professional Enhancement Continuous (Re-Certification) - not a practice in Brazil	This requirement encourages surgeons to engage in an ongoing process of learning and professional development, promoting excellence in the specialty over time.
Credibility of the Society	The surgical specialty society gains credibility by certifying professionals through a rigorous evaluation process. This reinforces the trust of the medical community and the public in society and its members. Even more so, if a periodic recertification process is implemented that guarantees patients that the specialist surgeon’s capacity remains proven and attested.

**Table 2:** Advantages of obtaining the Specialist Title by medical professionals.

Vantagens da obtenção do Título de Especialista pelos profissionais médicos	
Professional Recognition	It confers formal recognition of the physician's experience and competence in the specific area of expertise. This is valuable for building a solid reputation in the surgical field.
Credibility and Trust	It is a seal of approval that indicates to colleagues, patients, and healthcare institutions that the physician meets high standards of knowledge, skill, and professional ethics.
Market Differentiation	In a competitive professional landscape, having a specialist title makes the doctor stand out, providing a competitive advantage and potentially opening doors to professional opportunities.
Continuous Development	The process of obtaining the specialist title typically involves a commitment to continuous learning, encouraging the surgeon to stay up to date with the latest practices, technologies, and research in their field.
Access to Professional Networks	Many surgical societies provide opportunities for members to connect and collaborate with other professionals in the field. This can lead to valuable collaborations, mentorships, and networking.
Participation in Scientific Activities	Many societies promote conferences, workshops, and scientific activities that provide opportunities for presenting research, exchanging knowledge, and updating professionals.
Leadership Engagement	Skilled surgeons often can engage in leadership within society, participating in committees, boards, and contributing to the advancement of the specialty.
Ethical and Professional Standards	The certification process often encompasses both ethical and professional aspects, reinforcing the surgeon's commitment to high ethical standards in surgical practice. It also allows the qualified surgeon to present himself as a specialist in possession of a Specialist Qualification Registration (SQR), in view of his/her training and professionalism.
Best Job Opportunities	Having a specialist title can increase the chances of landing prominent positions in healthcare institutions, hospitals, or clinics that value specialization and certification.
Personal and Professional Satisfaction	In addition to the external benefits, obtaining the specialist title can provide a sense of personal and professional fulfillment, highlighting the doctor's dedication to their specialty.
Worldwide Recognition	The Brazilian Medical Association is affiliated to the World Medical Association (WMA), an international organization that represents physicians from all over the world and recognizes the titles generated by the specialties recognized by the Brazilian Medical Association (AMB).

 Here are some of the main advantages:

Since 1958, the AMB has granted the Specialist Title to physicians who have passed rigorous theoretical and practical evaluations with the objective of seeking scientific improvement and professional appreciation of physicians. Through its National Accreditation Commission, the AMB works on updating the titles, administering the necessary credits (AMB, 2021). The criteria for the public notices of the specialist titles exams must be in accordance with the requirements established in the agreement signed between the Federal Council of Medicine (CFM), the Brazilian Medical Association (AMB), and the National Commission for Medical Residency (CNRM, 2002), and with the Sufficiency Exam Regulation for Specialist Title or Certification of Area of Expertise of the AMB (2021).

For any of the 54 medical specialties recognized in Brazil, the Federal Council of Medicine, through its Regional Councils (CRM), can only register as specialists (granting the SQR) physicians who present at least one of these two documents: Certificate of Completion of Medical Residency accredited by the National Commission for Medical Residency (CNRM); Specialist title granted by the Brazilian Association or Society of the respective specialty, which is affiliated to the Brazilian Medical Association (AMB) and whose notice of the specialist title exam followed the rules of the AMB and is approved by it (FALK, 2006; AMB, 2021).

Residency and Specialist Title are certificates of a different nature, being independent. A doctor may have one or both, but one or the other entitles the specialist to register as such in a CRM. By determination of the AMB, to grant a Specialist Title only for an excellent curriculum or for proof of completion of Medical Residency is no longer allowed (FALK, 2006). Currently, it is always necessary to pass exams that involve a written test (cognitive assessment) and a practical test (assessment of technical skills and communication skills).

On the other hand, the certificates of completion of specialization courses have their recognition in the Academy, as well as a degree of importance in the labor market and in the curriculum. However, these certificates are not sufficient for the physician to be registered as a specialist in the Medical Councils (FALK, 2006).

Due to information disseminated in the press regarding Medical Specialist Titles issued by an entity not affiliated with the Brazilian Medical Association, it was clarified that:

- According to Decree No. 8,516/15, which establishes the rules for the recognition of medical specialties, and CFM Resolution No. 1,974/11, which regulates medical advertising, only those recognized as medical specialists with titles granted by CNRM or AMB can be recognized as medical specialists, for those who have been approved in specific specialty tests of the societies affiliated to the Brazilian Medical Association, and, in both cases, must be registered with the Regional Council of Medicine.
- On February 22, 2023, the Federal Regional Court of the 1st Region judged the action number 1026344-20.2020.4.01.3400, accepting the appeal filed by the Federal Council of Medicine and the Brazilian Medical Association, suspending the decision given in the first instance in favor of an association of doctors who offer postgraduate courses.
- In the ruling, the magistrate argued that Brazilian law does not authorize doctors to disclose postgraduate degrees, because, if they do so, they can “deceive eventual patients claiming to be specialists.”. And it is the role of the judiciary to protect the “collective right of the people not to be deceived by false medical experts.”.
- With that decision, it was once again forbidden for doctors with lato sensu postgraduate degrees to advertise themselves as specialists.

Thus, the Code of Medical Ethics, the rules of the Federal Council of Medicine (CFM) and now the Common Justice prohibit the physician from claiming to be a specialist, through business cards, prescriptions, office signs, health plans, etc., without having the Specialist Qualification Record issued by a CRM. (FALK, 2006).

Monitoring trends and understanding individual motivations are essential to better understand the panorama of medical education in Brazil.

Undergraduate medical studies in Brazil are characterized by terminality: the student receives full license to practice medicine upon completing the course at one of the country’s medical schools (BICA & KORNIS, 2020). 

A bottleneck was formed by the disproportionate growth of undergraduate vacancies in relation to medical residency (MR) vacancies, as shown in Medical Demography 2023 (SCHEFFER, 2023), with 41,805 undergraduate vacancies (77% in private courses) and 4,950 MR programs accredited in Brazil, authorized to train physicians in 55 specialties and 59 areas of expertise, recognized by the Joint Committee of Specialties (CME), composed of representatives of CNRM, CFM, and AMB. 

At the end of 2023, the opening of another 10,000 undergraduate medical vacancies was authorized, jointly by the Ministries of Education and Health (BRASIL, 2023), which were not computed in the current study of Medical Demography, which will make the bottleneck even greater for medical residency vacancies.

To the great astonishment of many medical professionals who had already graduated, contrary to what happened in previous years, most graduates were interested in going through MR. Even with the large increase in undergraduate vacancies across the country, there has been an annual decrease in the demand for first year residency vacancies (R1) in the programs, which in 2021 had 16,848 vacancies available.

On the one hand, there has been a huge expansion in the offer of lato sensu specialization courses and an increasing number of doctors advertising their work through social networks. On the other hand, we have seen a great deal of bad news, in the various types of media, about errors associated with harmful practices against patients, both by doctors and by professionals from various other areas, even non-health.

In this scenario, the Specialist Titles awarded by Medical Societies serve as an indicator of competence, quality, and professional ethics in medical practice. By establishing rigorous criteria for certification, requiring ongoing professional updating, and monitoring the practice of members, Medical Societies play a key role in protecting against professional malpractice and promoting high standards of health care.

While it is important for Medical Societies to maintain high standards of competence and quality in granting Specialist Titles, this requires a careful and thoughtful approach in defining the criteria for certification, considering the needs and realities of the healthcare system and medical practice. There is no justification for lowering the rigor of the evaluation for the specialist title granted by the AMB and its affiliated specialty societies. Specialists’ capacity and patients’ safety must always come first. Finally, faced with the uncontrolled dilemma of the number of physicians trained per year, we see the granting of the specialist title by Medical Societies as one of the last ethical and efficient barriers in the protection of the population. 

The Commission for the Specialist Title in General Surgery of the Brazilian College of Surgeons (COTECIG) has sought to improve its techniques for the preparation and review of items, the selection of clinical cases for the oral test, as well as the simulated stations, progressively improving the training of face-to-face and online evaluators. Thus, the commitment to excellence and the constant search for improvement are essential to ensure that obtaining the Specialist Title by the Brazilian College of Surgeons is recognized as an indication of proficiency and competence in the surgical area.
